# Lignocellulose as an insoluble fiber source in poultry nutrition: a review

**DOI:** 10.1186/s40104-021-00594-y

**Published:** 2021-06-17

**Authors:** Ilen Röhe, Jürgen Zentek

**Affiliations:** Department of Veterinary Medicine, Institute of Animal Nutrition, Freie Universität Berlin, 14195 Berlin, Germany

**Keywords:** Fiber, Growth performance, Gut health, Gut morphology, Lignocellulose, Microbiota, Nutrient digestibility, Poultry

## Abstract

Extensive research in recent years into the use of various fiber sources in poultry nutrition has led to the perception that dietary fiber is more than a simple diet diluent. Several studies showed that the feeding of insoluble fiber sources such as oat hulls, sunflower hulls or wood shavings may affect digestive physiology and function improving chickens health and growth performance. In this context, the effect of lignocellulose as an insoluble dietary fiber source is increasingly being investigated. Lignocellulose is a component of plant cell walls and consists mainly of the insoluble carbohydrate polymers cellulose and hemicelluloses as well as the phenolic polymer lignin. Lignocellulose is chemically and physicochemically different from other insoluble fiber sources and thus possibly has different effects on poultry compared to traditional fiber sources. Several studies investigated the effect of dietary lignocellulose on growth performance, nutrient digestibility, gastrointestinal tract development and intestinal microbiota in broilers and laying hens. Studies differed in terms of feed formulation and lignocellulose inclusion level as well as products of different suppliers were used. The results obtained are inconsistent; beneficial, indifferent or detrimental effects of feeding lignocellulose were observed, so that a final assessment of lignocellulose as a “novel” insoluble fiber source is difficult. This review article summarizes the results of studies in connection with the feeding of lignocellulose to poultry, compares them with those that have used other insoluble fiber sources and illuminates the possible mechanisms of action.

**Supplementary Information:**

The online version contains supplementary material available at 10.1186/s40104-021-00594-y.

## Introduction

In recent years, there have been increasing scientific reports that dietary fiber can have a positive effect on animal health and productivity. Fiber as feed component in poultry nutrition has traditionally been given little consideration as it has only a low nutritional value from a chemical point of view. However, due to its unique physicochemical properties, several studies showed that insoluble fiber sources may affect digestive tract development and function resulting in improved chicken health and growth performance [1–4]. Feeding experiments were mainly carried out with insoluble fiber sources that arise as by-products during industrial production such as oat hulls, sunflower hulls, soybean hulls, wheat bran or wood shavings. In the last decade, research has concentrated on the use of an “innovative” insoluble dietary fiber source, lignocellulose (LC). LC is a constituent of plant cell walls and thus the most abundant and bio-renewable biomass on earth [5]. Studies in farm and companion animals showed that dietary LC may have potential effects on digestive physiology and function [6–10]. This review gives a comprehensive overview of the effects of dietary LC in poultry. First, the physicochemical properties of LC are described and reference is made to methodological aspects of the incorporation of LC into feed, as this can have a decisive influence on the study results. Next, the results of studies on the effects of dietary LC on growth performance, nutrient digestibility, gastrointestinal tract development and intestinal microbiota are summarized and compared with those observed in feeding experiments using other insoluble fiber sources. In particular, the potential mode of action of insoluble dietary fiber on the digestive physiology of chickens is discussed. In addition, some considerations regarding future research directions and methodological challenges are presented and discussed.

### Chemical composition and physicochemical properties of LC

Dietary fiber comprises a significant part of plant feedstuffs and is chemically defined as the sum of non-starch polysaccharides (NSP) and lignin [11]. From a physiological point of view, dietary fiber comprises any polysaccharide and lignin that is not degraded by endogenous enzymes in the digestive tract, hence reaching the hindgut [12, 13]. Different types of plants contain different amounts and chemical structures of fibers with varying physical properties [14]. Therefore, fiber sources differ in their content of soluble and insoluble NSP. Fiber sources with high levels of insoluble NSP are for example LC, oat hulls, sunflower hulls or wheat bran, while sugar beet pulp or apple pomace contain higher concentrations of soluble NSP [3, 13]. Lignocellulosic biomass refers to plant dry matter of different origin and is mainly composed of the carbohydrate polymers cellulose and hemicelluloses as well as the phenolic polymer lignin [15, 16]. The proportional composition of carbohydrate and aromatic polymers of LC may vary depending on the type of lignocellulosic biomass used [17, 18]. LC applied in animal nutrition is usually derived from forest residues containing different proportions of hard and soft wood as well as bark. A recent study analyzed the chemical composition of three LC products used as fiber additives in animal feed [19]. Table 1 shows the chemical and physicochemical characteristics of these LC products compared to that of other insoluble fiber sources, in particular, oat hulls, sunflower hulls and wheat bran. The LC products showed a similar crude fiber content, but differences in the detergent fibers, which allow a rough assessment of the insoluble fractions of cellulose, hemicelluloses and lignin [13, 21]. Two products (LC2 and LC3) showed a similar cellulose, hemicelluloses and lignin content of ~ 415, ~ 150 and ~ 330 g/kg dry matter (DM), respectively, which are close to that reported by Zeitz et al. [22]. The LC1 product, however, contained significantly more lignin (~ 650 g/kg DM) and lower amounts of cellulose (~ 78 g/kg DM). All three LC products comprised high proportions of more than 90% insoluble fibers and only small amounts of soluble fibers [19, 22]. Similarly, oat hulls were mainly composed of insoluble dietary fiber [20] and contained primarily hemicelluloses and cellulose. Sunflower hulls showed slightly lower values for crude and detergent fiber, but the relative distribution of cellulose, hemicelluloses and lignin was similar when compared to LC2 and LC3. Sunflower hulls contained mostly insoluble fiber, but about twice as much soluble fiber compared to LC. Wheat bran had the lowest crude fiber content of all the fiber sources shown and was mainly composed of hemicelluloses. In addition, the proportion of soluble to insoluble dietary fibers in wheat bran was slightly higher compared to LC. With respect to the physicochemical properties, there is a positive correlation between dietary soluble fiber content and digesta viscosity in monogastric animals [13]. Due to the low proportion of soluble fibers such as pectins, insoluble dietary fiber sources have little effect on digesta viscosity [3, 14]. Another important physicochemical feature of dietary fiber is their hydration capacity, which can be characterized by the swelling capacity, the water holding and binding capacity [13]. The hydration capacity of a dietary component affects the bulking effect of digesta [23], which in turn could have consequences on digesta retention time and nutrient digestibility [24]. LC showed higher hydration capacities and significantly greater swelling properties compared to oat hulls, sunflower hulls and wheat bran [19, 20]. Finally but yet importantly, the particle size of a fiber source is another key characteristic, which may influence digestive function [23]. After processing and fiber breakdown, LC is a powdery material with an average particle size of 80 to 300 μm [22, 25]. This material can then be further processed, so that various LC products are commercially available, e.g. in powdery, crumbled or pelleted form. The particle size of other insoluble fiber sources is usually larger depending on the degree of grinding.

**Table 1:** Chemical and physicochemical characterization of different lignocellulose products in comparison with other insoluble fiber sources

Item	Unit	LC1^a,b^	LC2^a,b^	LC3^a,b^	Oat hulls^c^	Sunflower hulls^a^	Wheat bran^a^
Crude fiber	g/kg DM	579	559	561	302	535	145
Neutral detergent fiber (aNDF_OM_)		926	919	874	750	843	585
Acid detergent fiber (ADF_OM_)		728	757	737	357	679	181
Acid detergent lignin (ADL_OM_)		650	329	335	40	255	70
Total dietary fiber		953	945	949	762	897	612
Insoluble dietary fiber		942	933	938	754	871	579
Soluble dietary fiber		11	13	12	8	27	34
Water holding capacity	mL/g DM	5.21	4.63	7.43	3.9	4.35	5.51
Water binding capacity		7.29	6.30	6.35	N/A	5.88	5.09
Swelling property	%	205	150	185	2.1^d^	65	55

^a^ According to Slama et al. [19]; ^b^ Information on the LC product used, if specified, is given in additional file 1; ^c^ According to Jiménez-Moreno et al. [20]; ^d^ Indicated as mL/g DM

### Inclusion of insoluble fiber sources in experimental diets

**Fig. 1:**
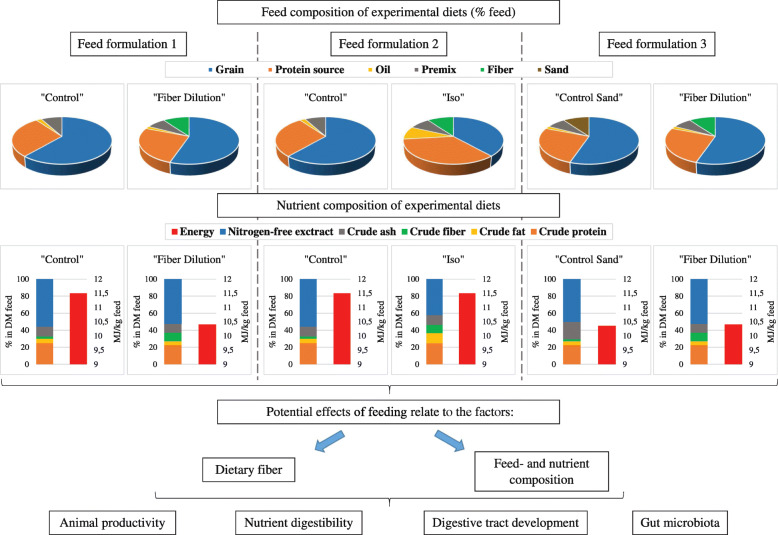
Overview of options to include an insoluble fiber source in experimental diets. Detailed legend: The feed and nutrient composition of three different options (feed formulation 1 to 3) to include an insoluble fiber source in experimental diets are presented. Feed formulations vary in terms of dietary fiber content as well as feed- and nutrient composition, which can have potential effects on the animal

In order to investigate the effect of dietary insoluble fiber in chickens, several feeding experiments were designed using different feed formulations. In principle, there are three different options to include fiber sources in diets, as displayed in Fig. 1. In “feed formulation 1”, a “control” feed is compared with a “fiber dilution” diet. A “control” poultry diet usually consists of grains, protein sources, plant oils and a premix. The nutrient composition of the “control” diet should meet the nutrient recommendations for chicken diets. The treatment diet “fiber dilution” is based on the “control” diet, but supplemented with the fiber source of interest. As insoluble fiber sources, such as LC, cellulose or wood shavings, have a low nutritive value, the energy and nutrient content of the diet is diluted by the added fiber source. In “feed formulation 2”, the same “control” diet is used, but the fiber containing diet is balanced to be isoenergetic and isonitrogenous. To achieve this, the “iso” diet must be formulated to have increased proportions of fat and protein at the expense of the carbohydrate source. Consequently, the “control” and the “iso” diet show remarkable differences in the feed- and nutrient composition, but show similar energy and protein concentrations. In “feed formulation 3”, a “control sand” and a “fiber dilution” diet is used. The “control sand” diet is based on the “control” feed, but contains a certain percentage of an insoluble ash source, e.g. sand or sepolite. The “fiber dilution” diet is based on the “control sand” diet, but the insoluble ash source is substituted by the insoluble fiber source of interest. Thus, the feed composition is similar with the exception of the components sand and fiber. As a result, the nutrient composition of both diets differs significantly only in terms of crude ash and crude fiber. In summary, the dietary inclusion of an insoluble fiber source is coupled with differences in the feed- and nutrient composition of the experimental diets. Therefore, the effects of feeding these diets can be attributed to both, the factor “dietary fiber” and the factor “feed and nutrient composition” (Fig. 1). Thus, alterations in animal productivity, nutrient digestibility, digestive tract development or gut microbiota might be related to differences in dietary fiber and/or feed and nutrient composition. Reference is made to this issue in the respective sections of this review.

### Impact of dietary LC on productivity of chickens

During the last decade, several studies examined the effect of feeding LC on the productivity of broilers (Table 2) and laying hens (Table 3). Productivity parameters include body weight (BW), average weight gain (AWG), average feed intake (AFI) or average daily feed intake (ADFI), feed conversion ratio (FCR), egg production (EP) and egg weight (EW). Studies differed in terms of feed formulation and LC inclusion level used. In most studies, dietary LC was supplemented on top of feed (“feed formulation 1”, Fig. 1); only few used experimental diets based on “feed formulation 2 and 3” (Fig. 1). Commonly, lower dietary LC inclusion levels in the range of 0.05% to 2% have been used, while few experiments were performed using relatively high concentrations of dietary LC of 5% to 15%.

**Table 2:** Impact of dietary lignocellulose on broiler growth performance

Feed^1^	Age, d	LC Inclusion^2^, %	Final BW^3^, g	AFI^4^, g	AWG^5^, g	FCR	Reference
1	21	0		1057	582	1.81^a^	[26]
		0.25		1049	596	1.75^a^	
		0.50		1033	615	1.67^b^	
		0.75		1030	618	1.66^b^	
	1–42	0		4016	1915^b^	2.1^a^	
		0.25		4086	2084^a^	1.96^b^	
		0.50		4030	2073^a^	1.94^b^	
		0.75		4156	2147^a^	1.93^b^	
1	1–42	0	2422		80.2	1.865	[27]
		0.25	2423		80.7	1.876	
		0.5	2436		81.0	1.854	
		1.0	2429		81.0	1.875	
3	7–14	0		161	69.2^b^	2.33^a^	[28]
		1		150	78.0^a^	1.92^b^	
		2		150	71.7^b^	2.09^ab^	
	14–21	0		326	128	2.55^a^	
		1		299	152	1.98^b^	
		2		291	145	2.03^b^	
1	1–10	0		294	277	1.059	[24]
		1		292	271	1.078	
		2		294	276	1.065	
	1–35	0		3770	2741	1.376	
		1		3778	2716	1.392	
		2		3797	2719	1.397	
1	1–21	0		1085	860	1.265^b^	[29]
		1		1054	815	1.297^a^	
1	1–7	0		129^b^	121^c^	1.07^a^	[30]
		0.4		132^b^	128^ab^	1.03^b^	
		0.6^6^		140^a^	132^a^	1.06^a^	
		0.6^7^		130^b^	126^bc^	1.03^b^	
	14–21	0		590	396^b^	1.49	
		0.4		588	402^b^	1.46	
		0.6^6^		621	432^a^	1.44	
		0.6^7^		594	403^b^	1.47	
	28–42	0	2428^b^	2295^a^	1284	1.79^a^	
		0.4	2423^b^	2084^b^	1245	1.67^b^	
		0.6^6^	2611^a^	2186^c^	1310	1.67^b^	
		0.6^7^	2495^b^	2182^bc^	1292	1.69^b^	
1	1–35	0	2431	3459	2390	1.42	[22]
		0.8^8^	2370	3386	2329	1.42	
		0.8^8^	2490	3452	2448	1.39	
1	13–25	0	1080^a^	0.250^9^	0.173^10^		[31]
		5	995^ab^	0.258^9^	0.164^10^		
		10	928^bc^	0.258^9^	0.153^10^		
		15	836^c^	0.270^9^	0.149^10^		
1	1–35	0	2154^bc^	1293^ab^	650	1.58^a,11^	[32]
		0.05	2201^b^	1266^bc^	631	1.51^b,11^	
		0.1	2305^a^	1314^a^	667	1.50^b,11^	
		0.2	2142^c^	1266^c^	626	1.55^ab,11^	
2	70–91	0.8	1658	899	454	1.98	[25]
		5	1639	992	467	2.15	
		10	1618	905	432	2.10	

^1^ Feed formulation according to Fig. 1; ^2^ Information on the LC product used, if specified, is given in additional file 1;^3^ BW = body weight; ^4^AFI = average feed intake; ^5^ AWG = average weight gain; ^6^ LC was included in the diet at the expense of 0.3% soybean meal and 0.3% maize; ^7^ LC was included in the diet at the expense of 0.6% soybean meal; ^8^ Two different lignocellulose products were compared; ^9^ Indicated as average daily feed intake in (g/d)/BW; ^10^ Indicated as average daily gain in g/d/BW; ^11^ Indicated as g weight gain/g feed consumption; ^a,b,c^Means with different superscripts are significantly different

**Table 3:** Impact of dietary lignocellulose on hen productivity

Species	Feed^1^	Age, week	LC inclusion^2^, %	Final BW^3^, g	ADFI^4^, g	EP^5^, %	EW^6^, g	Reference
Laying hen	1	30	0		126	94.7	65	[33]
			1		131	95.5	66	
		36	0		144	93.4	66	
			1		144	93.7	66	
Pullet	1	8–18	0	1580				[34]
			1	1678				
Laying hen	1	22–31	0	1902				[34]
			0.8	1919				
Pullet	1	1–8	0	766	50.6			[35]
			1	776	52.4			
			2	764	51.6			
Laying hen	1	18–38	0	1655^c^	96.3^b^	78.0^c^	55.3^c^	[36]
			0.05	1693^b^	98.0^a^	80.8^b^	56.4^b^	
			0.1	1719^a^	99.2^a^	81.8^a^	57.3^a^	
			0.2	1636^c^	95.4^b^	78.6^d^	54.4^d^	
Broiler breeder hen	3	43–55	0	~4500^a7^	174^a^	56.9^b^	69.9	[37]
			3	~4300^b7^	172^b^	62.9^a^	69.2	
Dual purpose hen	1	1–22	0	1835^a^	61.1			[38]
			10	1694^b^	61.2			
		23–52	0	1996^a^	101^b^	63.4^b^	60.6^a^	
			10	1791^b^	107^a^	72.4^a^	58.0^b^	

^1^ Feed formulation according to Fig. 1; ^2^ Information on the LC product used, if specified, is given in additional file 1; ^3^ BW = body weight; ^4^ ADFI = average daily feed intake; ^5^ EP = egg production; ^6^ EW = egg weight; ^7^ Data on BW are estimated because they were taken from a figure; ^a,b,c^ Means with different superscripts are significantly different

Results obtained in broiler trials using relatively low dietary concentrations of LC are contradictory (Table 2). The feeding of diets supplemented with 0.25% to 2% LC positively affected the FCR of broilers due to an increase in weight gain [26, 28]. In contrast, broilers fed diets supplemented with 1% LC had an impaired FCR compared to those receiving the control diet [29]. However, several studies using similar LC inclusion levels showed no impact of dietary LC on broiler growth performance [22, 24, 27]. Results of a recent study demonstrated that dietary LC concentrations of 0.05% to 0.1% improved FCR of broilers, while the supplementation of 0.2% LC showed no effect on FCR [32]. Broilers fed 0.6% LC, which was added at the expense of soybean meal and corn, showed higher BW after 42 d of feeding compared to those fed the control and 0.4% LC [30]. Interestingly, the feeding of the same LC concentration of 0.6%, but which was added at the expense of soybean meal only, did not affect final BW of broilers [30] suggesting that the feed composition had a greater impact on BW development than the LC addition. Broilers fed diets diluted with relatively high concentrations of LC of 5% to 15% showed a marked decrease in average daily gain with increasing concentrations of dietary LC, while feed intake tended to increase with increasing concentrations of LC [31]. On the contrary, broiler productivity seems to be unaffected by relatively high dietary LC inclusion levels up to 10% when diets were composed to be isoenergetic and isonitrogenous [25, 39].

With respect to feeding experiments with commercial hybrid pullets and laying hens (Table 3), most studies showed that growth and laying performance were not affected by dietary LC inclusion levels of 0.8% to 2% [33–35]. However, Sozcu and Ipek [36] demonstrated that the supplementation of 0.05% and 0.1% dietary LC increased BW, ADFI, EP, and EW of laying hens between 18 to 38 weeks of age compared to hens fed the control diet. A further increase in the dietary LC concentration to 0.2%, however, led to a decrease in EP and EW [36]. In two other studies, the effect of dietary LC was investigated in broiler breeder hens [37] during the laying phase (43 to 55 weeks of age) and in dual purpose hens [38] during the growing (1–22 weeks of age) and laying period (23–52 weeks of age). Broiler breeder and dual purpose hens tended to overconsume feed leading to an increased body fat content, which in turn might be related to the observed lower productive efficiency [40–42]. Thus, the hypothesis in both studies was that BW and body fat percentage of hens can be reduced by feeding a nutrient-reduced LC-containing diet and that this is accompanied with an improved reproductive performance [37, 38]. The results showed that dietary LC reduced BW, body fat content [38] and abdominal fat weight of hens [37], which was directly associated with an improved laying performance.

In principle, due to the use of different feed formulations and inclusion levels, it is difficult to make a conclusive statement about the effect of LC on chicken productivity. Few studies showed that similar insoluble fiber sources such as cellulose or wood shavings could have a positive impact on broiler growth performance [43, 44]. It was suggested that a combination of improved gut function and enhanced nutrient digestibility was responsible for observed beneficial effects [43, 44]. With regard to dietary LC and lower inclusion levels, it was also hypothesized that digestive physiology and nutrient digestibility might be affected leading to improved growth performance [26, 28, 30]. However, data on the effects of dietary LC on digestive tract development and nutrient digestibility were inconclusive, as described later. Studies generally showed that animal productivity was impaired when diets were supplemented with higher dietary LC concentrations. This observation is explained by the fact that the energy- and nutrient content of diets was considerably reduced by the LC inclusion (“feed formulation 1”, Fig. 1) resulting in a lower energy and nutrient intake of chickens impairing growth performance. However, animal productivity seems to be not affected when higher concentrations of LC are included in isoenergetic and isonitrogenious diets (“feed formulation 2”, Fig. 1). This phenomenon is already known from former studies, which showed that the crude fiber concentration did not influence growth performance unless it affected the energy content in the diets [45–47]. Chickens usually have the ability to cover their metabolic energy requirement to a certain degree by increasing or decreasing the feed consumption [48, 49]. Chickens receiving diets diluted by sand or oat hulls up to 20% showed an increased feed intake resulted in a similar energy intake and average daily gain in comparison to those receiving an undiluted control diet [49, 50]. With respect to higher dietary inclusion levels of powdery LC, this regulatory mechanism seem to be partially restricted [31, 38], which might be attributed to the physical form of fine LC fibers and its physical bulking effect [37, 38]. Accordingly, the use of higher concentrations of powdery LC in feed for broiler breeder and dual purpose hens, might be a reasonable dietary strategy to control feed intake and weight gain ensuring an optimal productive performance.

### Impact of dietary LC on the nutrient digestibility in chickens

Data regarding the effect of dietary LC on nutrient digestibility in chickens are scarce displaying no clear picture. A direct comparison of results is difficult: on the one hand, diets differed in their feed composition and nutrient content; on the other hand, different dietary LC concentrations were used. The feeding of isoenergetic and isonitrogenous diets containing 0.8% LC increased the true digestibility of protein as well as the apparent and true dietary amino acid digestibility in roosters compared to those fed the control diet [51]. Similarly, the same authors observed that the apparent protein digestibility was increased by 5.5% in broilers fed 0.8% dietary LC compared to those receiving the control diet [52]. In contrast, the supplementation of 1% or 2% dietary LC did not affect protein and gross energy digestibility in broilers [24, 29]. Feeding of isoenergetic and isonitrogenous diets with higher LC inclusion levels of 5% and 10% led to a decrease in the apparent ileal digestibility of crude protein and apparent excreta digestibility of organic matter and gross energy while the total tract digestibility of ether extract was not affected [25]. The apparent ileal fat digestibility and total tract digestibility of total fatty acids in broilers was also not influenced by the supplementation of 0.25% and 0.5% LC while the feeding of 1% LC resulted in an increased apparent fat digestibility [53].

If dietary LC has an impact on the digestibility of nutrients, either beneficial or detrimental, the question arises as to how LC might affect the digestive physiology of chickens. Regarding the beneficial effects, it is well known that the feeding of structural components, such as coarse fiber particles, may stimulate digestive function, which is associated with an improved nutrient digestibility [23, 54]. LC consists of very small fiber particles and thus it remains unclear whether dietary LC may affect the digestive physiology and thus the nutrient digestibility in chickens, as discussed later. Another positive effect of feeding LC might be related to the fat digestibility. Jiménez-Moreno et al. [55] speculated that dietary cellulose might have an effect on micelle formation and lipid absorption, enhancing bile acids recycling and fat absorption. Further research is needed in order to evaluate this hypothesis. With respect to the potential adverse effects, it was assumed that dietary LC might have an abrasive effect on the intestinal mucosa [27, 30], thus enhancing endogenous amino acid losses. In this regard, Kluth and Rodehutscord [56] showed that the feeding of increasing concentrations of cellulose up to 8% significantly elevated the inevitable losses of crude protein and amino acids in broilers. Whether this observation also applies to the feeding of increasing concentrations of LC requires further clarification. In summary, based on the studies carried out so far, no statement can be made about whether LC has an impact on nutrient digestibility in chickens.

### Impact of dietary LC on gastrointestinal tract development, intestinal morphology and excreta characteristics of chickens

Few studies exist evaluating the effect of dietary LC on the gastrointestinal tract development and digestive physiology of broilers and laying hens. Investigations were focused on the gastrointestinal gross morphology, the intestinal histomorphology, and digesta as well as excreta characteristics.

### Effect on gizzard development and function

In broilers, most studies showed that the feeding of lower dietary LC concentrations up to 2% did not affect the relative weight of the gizzard [22, 24, 28–30]. However, feeding of isoenergetic and isonitrogenous diets containing 5% LC resulted in an increased gizzard weight of slow growing broilers [39]. Similarly, the relative gizzard weight was affected by feeding LC in laying hens and pullets [34]. Pullets fed 1% LC over a period of 10 weeks showed increased relative weights of the gizzard. Moreover, laying hens, aged 31 weeks, developed heavier gizzards when fed diets diluted with 0.8% LC after 12 weeks of feeding [34]. Interestingly, these effects were not observed in chickens that received these diets for a shorter period, suggesting a time-dependent effect of LC. Studies in quails also showed that the feeding of isoenergetic and isonitrogenous diets containing 3% LC increased the relative gizzard weights [57]. Few studies investigated the effect of dietary LC on gizzard pH showing conflicting results. The feeding of diets supplemented with 0.4% and 0.6% LC decreased the gizzard pH of broilers [30], while the feeding of 0.8%, 1% and 2% dietary LC had no impact on gizzard pH [22, 24]. Broilers fed diets containing 0.05% to 0.2% LC showed also a similar gizzard pH compared to those fed the control diet [32].

**Fig. 2:**
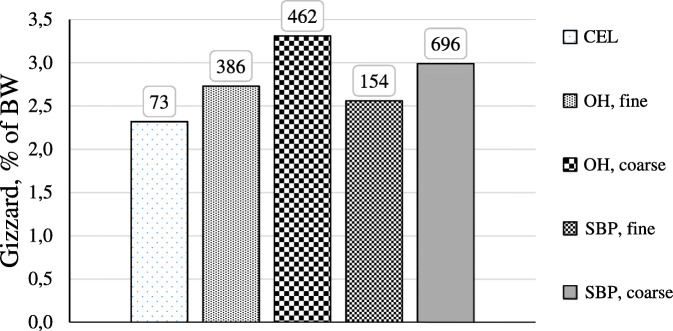
Impact of dietary fiber and particle size on the gizzard weight of broilers. Detailed legend: Effect of type and particle size of dietary fiber on the relative gizzard weight of broilers according to Jiménez-Moreno et al. [55]; CEL = cellulose-fed broilers; OH = oat hulls-fed broilers; SBP = sugar beet pulp-fed broilers; the geometric mean diameter of diets is indicated above the respective bar

In general, coarsely ground fiber sources such as oat, soybean and pea hulls, or wood shavings, containing primarily insoluble NSP, are known to increase gizzard size and weight [43, 58, 59]. An increased gizzard weight might be an indicator of enhanced gizzard function [23, 54, 60, 61]. Several feeding experiments using different coarsely ground fiber sources showed that an increased gizzard weight was accompanied with a lower gizzard pH [59, 61–63] suggesting an enhanced proventricular secretion of hydrochloric acid. Moreover, an increased gizzard activity is related to an increased gastrointestinal reflux and pancreatic enzyme secretion [64–67]. Furthermore, the feed passage rate might be affected by feeding structural fiber components improving nutrient digestibility and growth performance of chickens. In this regard, it is generally accepted that the feeding of coarsely ground, insoluble fibers increases the feed passage rate [23]. However, in poultry it is suggested that the feeding of moderate amounts of structural dietary fiber reduces the digesta transit time, as fiber particles may accumulate in the gizzard [23, 58]. A recent study proved that digesta transit time of broilers was not affected by feeding diets containing finely ground LC or oat hulls [29]. The question arises whether the fiber inclusion itself, the particle size of the fiber source or a combination of both factors are responsible for observed effects on gizzard development and digestive function. In this regard, Jiménez-Moreno et al. [55] investigated the impact of type and particle size of dietary fiber on gizzard weight of broilers (Fig. 2). Diets contained different fiber sources, in particular cellulose, oat hulls and sugar beet pulp at inclusion levels of 3%. Furthermore, diets differed in terms of particle size distribution indicated by different geometric mean diameters (GMD). Results indicated that broilers fed diets showing the highest GMD, namely coarsely ground oat hulls and sugar beet pulp, developed the greatest gizzard weights, while broilers fed diets having the lowest GMD, cellulose and finely ground sugar beet pulp, showed the lowest gizzard weights (Fig. 2). It was concluded that dietary cellulose did not stimulate gizzard function due its lack of physical structure [55]. Thus, it seems obvious that particle size of the fiber source, rather than fiber inclusion itself, is the determining factor that stimulates gizzard development. In this regard, it has been suggested that feed particles should be at least larger than 1 mm to enhance gizzard development [54, 68]. Based on this, it can therefore be assumed that fine-fiber LC, similar to cellulose, has little effect on gizzard development and function. In this context, it would be interesting to investigate whether the physical form or the macrostructure of LC might have an impact on gizzard development. Therefore, future studies should investigate the effects of the physical form of LC and that of the overall feed structure on digestive physiology in chickens.

### Effect on the intestinal gross morphology

Only few studies investigated the impact of dietary LC on the development of the small and large intestine, but most of them showed no effect of LC feeding on the intestinal gross morphology. In broilers, the feeding of relatively low dietary LC inclusion levels of 0.4% and 0.6% did not affect the relative weight of the intestine [30]. In agreement with this, broilers fed diets supplemented with 1% LC had similar relative duodenal, jejunal and cecal weights compared to those fed the control diets [29]. Relative lengths of small intestinal segments were also comparable between broilers fed 0.25% to 1% dietary LC and those offered the control diet [69]. Similarly, the absolute length of the small intestine and cecum of broilers was not influenced by feeding isoenergetic and isonitrogenous diets containing 2% and 4% LC. In another study, the feeding of isoenergetic and isonitrogenous diets containing 3% LC increased the relative weight of the jejunum and ileum of quails [57]. Pullets fed 1% LC over a period of 10 weeks showed comparable relative weights of the small intestine compared to those fed the control diet [34]. The feeding of diets supplemented with LC concentrations of 1% to 2% also showed no impact on the absolute cecal weight and length of laying hens [35] nor on the relative cecal weight of pullets [34]. The feeding of isoenergetic and isonitrogenous diets containing higher concentrations of LC up to 5% also had no impact on the absolute length of the small intestine and cecum of slow growing broilers [39]. In contrast, the feeding of energy- and nutrient reduced diets containing 10% LC, led, in relation to the BW, to increased weights of the small and large intestine [70].

Reasons for alterations in length or weight of intestinal organs due to feeding insoluble fiber sources are not fully understood. In general, it is supposed that an increase in the intestinal size and length but also an enlargement of the intestinal mucosa contributes to a higher intestinal weight [71]. Several studies demonstrated that chickens fed different insoluble fiber sources at varying inclusion levels showed increased relative digestive tract weights [63, 70, 72] implying a fiber-related effect on intestinal organ development [63, 72]. However, in those studies dietary fiber inclusion also led to a decrease of the chicken’s BW [63, 70, 72]. Therefore, the hypothesis that dietary fiber influenced organ weight development in those chickens is not valid as data on intestinal weight and length are related to the BW. Only considering studies in which chickens had similar empty BW, results on the effect of fiber on intestinal tract development are conflicting. On the one hand, it has been reported that feeding of isoenergetic and isonitrogenous diets containing 3% insoluble fiber sources, such as oat and soybean hulls, increased the digestive tract weight of broilers [59, 61, 73, 74]. On the other hand, the feeding of isoenergetic and isonitrogenous diets containing 3% oat hulls, soybean hulls or cellulose did not affect the relative weight of intestinal organs [55, 75, 76]. Similarly, the feeding of diets, supplemented with 10% oat hulls or cellulose, did also not affect the relative weight of intestine of broilers [77, 78]. Regarding possible fiber-effects on intestinal tract development, it was suggested that an enlargement of the digestive tract might be a consequence of physical distension caused by luminal swelling of the ingested fiber sources [59, 63, 72]. Further research is needed in order to clarify whether and why dietary insoluble fiber may have an impact on gut gross morphology.

### Effects on the intestinal mucosal development

Few studies showed that dietary LC might affect the morphology of the intestinal mucosa of chickens. Sarikahn et al. [26] showed that ileal villus height and villus height to crypt depth ratio were increased in broilers fed diets containing 0.25%, 0.5% and 0.75% LC. Similarly, broilers receiving diets supplemented with up to 2% LC had an increased jejunal villus height, villus apparent surface area and villus height to crypt depth ratio compared to those fed the control diet [32]. In contrast, duodenal and jejunal villus height and crypt depth were reduced in broilers fed diets supplemented with 0.5% LC while the inclusion of 1% LC showed no effect on villus morphology [69]. Interestingly, a different observation was made in the ileum of those chickens as increasing concentrations of dietary LC led to an increase in villus height and crypt depth [69]. The feeding of 0.6% LC, which was included in the diet at the expense of soybean meal, resulted in an increased villus height and width as well as crypt depth in the duodenum, jejunum and ileum of broilers [30]. However, the same inclusion level of 0.6% dietary LC, supplemented at the expense of 0.3% soybean meal and 0.3% corn, did not affect duodenal and jejunal villus height and villus width of broilers [30] implying that intestinal mucosal development was influenced by feed composition rather than dietary LC inclusion. Abdollahi et al. [29] showed that the supplementation of 1% dietary LC had no impact on histomorphological parameters in the duodenum and jejunum of broilers. In laying hens, the feeding of 0.05% and 0.1% dietary LC increased jejunal villus height and width, villus apparent surface area and villus height to crypt depth ratio, while a further increase in dietary LC concentration of 0.2% decreased observed histomorphological parameters [36]. Dual purpose hens fed diets supplemented with 10% LC showed an enhanced mucosal development of the colorectum indicated by a greater villus area and a higher villus and crypt mucosal enlargement factor [70]. Interestingly, the colorectal villus surface was negatively correlated with the short chain fatty acid (SCFA) concentration in the cecum of hens [70]. Another study showed that the feeding of 3% dietary LC included in isoenergetic and isonitrogenous diets increased the villus height and villus height to crypt depth ratio in the jejunum and ileum of quails [57].

An enlargement of the intestinal surface area due to longer or increased numbers of intestinal villi is generally associated with an increased intestinal nutrient absorption [79, 80] and thus an improved nutrient utilization. However, the development of the intestinal microarchitecture strongly depends, among other things, on the concentration of enteral nutrients and thus on the nutrient content of the diet [81, 82]. It has been suggested that chickens fed high-fiber diets suffer from a nutrient deficiency and thus try to enhance nutrient and bacterial metabolite absorption by increasing the mucosal surface area [70, 82, 83]. Thus, it has to be emphasized that both the dietary nutrient content and/or the fiber inclusion might affect the mucosal development of the intestine (Fig. 1). However, effects on intestinal mucosal development were also observed in studies using isoenergetic and isonitrogenous diets or lower dietary fiber inclusion levels [26, 30, 74], so that similar enteral nutrient concentrations can be expected. The potential mode of action of dietary insoluble fiber on intestinal mucosal development in chickens is still unknown. Whether specific chemical and physicochemical properties of the fiber source or changes in the intestinal microbiota due to fiber feeding could be responsible requires further clarification.

### Effects on excreta quality

Studies in broilers indicated that dietary LC inclusion might have a positive effect on litter quality. Litter moisture content was lower in broilers fed diets supplemented with 0.6%, 0.8%, 1%, and 2% LC compared to litter of control-fed broilers [24, 30, 52]. Similarly, litter moisture content was also reduced in quails fed 3% dietary LC [57]. The litter DM content usually correlates with the DM content of the excreta. Consistent with the latter, laying hens fed 10% LC for 52 weeks had a higher excreta DM content at 10, 17 and 22 weeks of age compared to those fed the control diet [70]. However, studies in broilers showed no impact of dietary LC on excreta scoring or excreta DM [22, 25].

In comparison with other insoluble fiber sources, LC has a moderate to high water holding capacity [19]. It has been speculated that the water holding capacity and the digesta retention time might be increased in LC fed chickens resulting in increased luminal water absorption and higher excreta DM content [24]. In addition to the hydration capacity, digesta- and excreta DM might be also affected by the particle size of the fed fiber source. Excreta score was improved in broilers fed coarsely ground wood shavings at a ratio of 6:100 (w/w), while finely ground cellulose- and control-fed broilers showed comparable excreta scores [43]. Authors speculated that coarsely ground fibers might hold larger amounts of water reducing the solubilisation of NSP than finely ground fiber particles [43]. Further research is needed in order to clarify whether and why insoluble fiber sources might reduce excreta DM and thus improve litter quality.

### Impact of dietary LC on the gut microbiota

It is well known that dietary fiber can modulate the gut microbiota in humans and animals, which in turn might have consequences on the intestinal health [84, 85]. Thus, few studies evaluated the impact of dietary LC on the intestinal microbiota in chickens. Alterations in the microbial composition can be accompanied with changes in the production of bacterial metabolites; vice versa, shifts of the intestinal bacterial metabolite profile are a clear indicator for a modification of the composition and activity of intestinal bacteria. Therefore, investigations were focused on both, bacteria residing in the avian intestinal tract and the concentration of intestinal bacterial metabolites. Table 4 shows the impact of dietary LC on the concentration of SCFA in the intestine of chickens. In general, results are conflicting, which may be explained by differences in the used study design, in particular regarding the used feed formulation, LC inclusion level and LC product.

**Table 4:** Impact of dietary lignocellulose on the concentration of intestinal short chain fatty acids (SCFA)

Species	Feed^1^	Part^2^	LC inclusion^3^, %	Unit	Total SCFA	Acetate	Propionate	Butyrate	Reference
Broiler	1	Ile	0	μmol/g	53^b^	9.16^bc^	3.78^b^	4.54	[27]
			0.25		61.1^ab^	7.16^c^	9.72^ab^	5.22	
			0.5		86.3^a^	20^a^	17.4^a^	5.33	
			1		69.6^ab^	18.3^ab^	9.18^ab^	4.54	
Broiler	1	Cec	0	μmol/g	124^b^	29.3	22.0	6.70	[27]
			0.25		150^ab^	30.1	25	6.58	
			0.5		162^a^	32.8	26.1	6.36	
			1		129^b^	26	23.8	5.79	
Broiler	1	Cec	0	μmol/g	126	101	5.98	17.8^a^	[22]
			0.8^4^		127	104	4.92	17.0^a^	
			0.8^4^		119	99	5.28	13.1^b^	
Broiler	2	Cec	0	mmol/L		5.20	1.82	0.80	[86]
			2			4.28	1.30	0.64	
			4			4.09	1.50	0.58	
Broiler	2	Cec	0.8	μmol/g	56.8^a^	38.1	8.89^a^	8.45^a^	[25]
			5		58.2^a^	39.1	9.83^a^	8.04^a^	
			10		44.9^b^	33.9	4.82^b^	5.17^b^	
Laying hen	1	Cec	0	mmol/100 g		2.42^b^	0.62^b^	0.19^b^	[35]
			1			4.07^a^	0.96^a^	0.28^a^	
			2			3.01^b^	0.72^b^	0.20^b^	
Laying hen	1	Cec	0	μmol/g	47.5^a^	37.5^a^	3.79^a^	4.83	[70]
			10		29.2^b^	23.2^b^	2.19^b^	2.89	

^1^ Feed formulation according to Fig. 1; ^2^ Intestinal part, Ile = Ileum, Cec = Cecum; ^3^ Information on the LC product used, if specified, is given in additional file 1; ^4^Two different lignocellulose products were compared; ^a,b,c^ Means with different superscripts are significantly different

Few studies used the same LC product, but different LC inclusion levels and feed formulations [25, 27, 30, 70]. The feeding of diets supplemented with LC at relatively low inclusion levels of 0.25% to 0.6% reduced counts of *Escherichia coli* and *Clostridium perfringens* and increased those of *Bifidobacterium* spp. and lactic acid bacteria in the ileum and cecum of broilers [30]. Similarly, ileal counts of *Lactobacillus* spp. as well as ileal and cecal counts of *Bifidobacterium* spp. were elevated in broilers fed diets supplemented with 0.25%, 0.5% and 1% LC [27]. Ileal and cecal counts of *Escherichia coli* and *Clostridium* spp. were also reduced in broilers receiving 0.25% and 0.5% dietary LC [27]. In the same experiment, however, the intestinal SCFA profile was generally not affected by LC feeding (Table 4), with the exception of broilers receiving 0.5% LC, which showed increased total SCFA concentrations in the ileum and cecum [27]. Two further studies evaluated the effect of relatively high concentrations of dietary LC on bacterial composition and metabolites [25, 70]. The feeding of diets diluted with 10% dietary LC had generally no impact on cecal microbial composition in dual pupose hens, but reduced the cecal concentration of SCFAs and ammonia [70]. Similarly, cecal bacterial metabolites were reduced in broilers fed isoenergetic and isonitrogenous diets containing 10% LC [25]. Moreover, increasing concentrations of dietary LC decreased counts of Escherichia/Hafnia/Shigella [25]. Four recent studies using a potential more fermentable LC product also displayed conflicting results regarding the effect of dietary LC on the gut microbiota in broilers and laying hens [22, 24, 35, 86]. The feeding of isoenergetic, isonitrogenous diets containing 2% and 4% LC increased the cecal microbial diversity and the abundance of butyrate-producing bacteria in free-range chickens, while the luminal concentration of butyrate, acetate and propionate was not affected [86]. In contrast, broilers and laying hens fed diets which were supplemented with 0.8% and 1% LC, showed no alterations in the overall cecal microbial diversity [22, 35]. Sun at al [35]. showed that the feeding of 1% LC increased the relative abundance of lactate- and butyrate-producing bacteria in the cecum of laying hens, which was accompanied with higher concentrations of cecal SCFAs. In contrast, the total amount of cecal SCFAs was not affected in broilers fed diets supplemented with 0.8% of the same LC product [22]. Moreover, LC-fed broilers had a lower cecal abundance of the bacteria families Ruminococcaceae and Lactobacillaceae as well as a higher abundance of Clostridiaceae, Enterobacteriaceae, Peptostreptococcaceae and Erysipelotrichaceae [22]. Diets diluted with 1% and 2% LC had in general no effect on detected bacteria except that counts of *Ruminococcus* spp. were increased and those of *Clostridium* spp. reduced in the cecum of broilers fed 2% LC [24].

Based on the studies carried out so far, no uniform picture can be drawn as to whether and to what extent dietary LC influences the intestinal microbiota in chickens. It is generally agreed that insoluble fiber sources such as LC, cellulose or wood shavings, are not extensively degraded by intestinal bacteria residing in the avian digestive tract [14, 85, 87]. On the one hand, this is due to the anatomical peculiarities of the chicken’s digestive tract, which is relatively short, resulting in a short feed passage rate. In addition, several studies suggest that only small and soluble fiber fractions can enter the cecum [66, 88, 89], which appears to be the main site for bacterial fermentation of fiber in chickens [90, 91]. On the other hand, there is evidence that the cellulolytic activity of bacteria in the chicken’s hindgut seems to be low [92–94]. Consequently, it is assumed that the impact of insoluble fiber on intestinal bacterial composition and activity appears to be minimal [14, 95]. However, some authors speculated that LC could be fermented in the cecum of chickens as intestinal bacterial composition or SCFA profile had changed due to dietary LC inclusion [22, 27, 35]. Furthermore, some studies used an “eubiotic” LC product, which might have a higher susceptibility to microbial fermentation than the standard LC product [22, 35, 86, 96, 97]. Moreover, it was suggested that dietary LC may have an abrasive effect on the intestinal mucosa and adhering bacteria [27, 30] or that phenolic compounds of lignin exhibit antimicrobial properties [22, 27].

The major problem in answering the question of whether dietary insoluble fibers generally have an effect on the intestinal microbiota of chickens refers to the experimental diets chosen to study that effect. With reference to Fig. 1, most studies that investigated the effect of insoluble fiber on gut microbiota, chose experimental diets based on “feed formulation 1”, and a few those based on “feed formulation 2”. Depending on the amount of dietary fiber added, there are corresponding changes in the nutrient composition between the control and the fiber containing diet (Fig. 1). Alterations in the nutritional composition of the feed result in changes of the amount of substrate that reaches the large intestine and can be fermented by resident bacteria [25]. As a consequence, changes in the dietary nutrient composition may influence the gut microbiota and bacterial fermentation pathways [25], making it difficult to distinguish between nutrient composition- and fiber related effects. The best way to study the effect of insoluble fiber on gut microbiota is possibly to use feed variants according to “feed formulation 3” (Fig. 1). Feed and nutrient composition of control and fiber diets are very similar, although it cannot be ruled out that even the inclusion of an insoluble ash sources might affect the gut microbiota.

## Conclusions

In conclusion, several studies were performed in order to evaluate the effect of dietary LC as an insoluble fiber source in poultry nutrition. Data on the impact of LC on growth performance, nutrient digestibility, digestive tract development and gut microbiota in chickens are inconsistent and do not allow a conclusive assessment. One of the reasons for this is that a direct comparison of results is difficult as studies differed in terms of feed formulations, LC inclusion levels and LC products. In future research, more attention should be paid to the type of feed formulation used in order to better distinguish the effects of dietary fiber from those of the feed and nutrient composition. In addition, the mode of action of LC in the digestive tract should be examined more closely, with particular reference to its chemical and physicochemical properties.

## Supplementary Information

**Additional file 1.** Overview on LC products used in the different studies. Description of data: Additional information on LC used in the different studies including supplier information and product name.

## Data Availability

Not applicable.

## Abbreviations

LC: Lignocellulose
NSP: Non-starch polysaccharides
BW: Body weight
AWG: Average weight gain
AFI: Average feed intake
ADFI: Average daily feed intake
FCR: Feed conversion ratio
EP: Egg production
EW: Egg weight
DM: Dry matter
GMD: Geometric mean diameter
SCFA: Short chain fatty acids
