# Correction to: Lignocellulose as an insoluble fiber source in poultry nutrition: a review

**DOI:** 10.1186/s40104-021-00623-w

**Published:** 2021-08-19

**Authors:** Ilen Röhe, Jürgen Zentek

**Affiliations:** Department of Veterinary Medicine, Institute of Animal Nutrition, Freie Universität Berlin, 14195 Berlin, Germany

**Correction to: J Anim Sci Biotechnol 12, 82 (2021)**

**https://doi.org/10.1186/s40104-021-00594-y**

Following publication of the original article [1], the Additional table 1 was noticed to be incorrectly formatted. The correct Additional table 1 is included in this Correction below.

The original paper has been updated.

## Supplementary Information

**Additional file 1: Table 1.** Overview on LC products used in the different studies.
