# Supplementary material for: Lignocellulose as an insoluble fiber source in poultry nutrition: a review
Source: J Anim Sci Biotechnol. 2021 Jun 17;12:82. doi: 10.1186/s40104-021-00594-y (PMC8212492; doi:10.1186/s40104-021-00594-y)
Supplement: Supplementary file 1 — Additional file 1. Overview on LC products used in the different studies. Description of data: Additional information on LC used in the different studies including supplier information and product name. [file 40104_2021_594_MOESM1_ESM.docx]

Additional table 1. Overview on LC products used in the different studies

|  | LC source | |
| --- | --- | --- |
| Reference | Supplier | Product |
| [19] | unspecified | unspecified |
| [22] | Agromed Austria GmbH | FibreCell® |
|  |  | OptiCell® |
| [24] | Agromed Austria GmbH | OptiCell® |
| [25] | J. RETTENMAIER & SÖHNE GmbH + Co KG | ARBOCEL® R |
| [26] | J. RETTENMAIER & SÖHNE GmbH + Co KG | unspecified |
| [27] | J. RETTENMAIER & SÖHNE GmbH + Co KG | ARBOCEL® RC |
| [28] | J. RETTENMAIER & SÖHNE GmbH + Co KG | ARBOCEL® RC FINE |
| [29] | Agromed Austria GmbH | OptiCell® |
| [30] | J. RETTENMAIER & SÖHNE GmbH + Co KG | ARBOCEL® R |
| [31] | J. RETTENMAIER & SÖHNE GmbH + Co KG | ARBOCEL® RC FINE |
| [32] | GLOBAL NUTRITECH® | LIGNOCHAR® |
| [33] | J. RETTENMAIER & SÖHNE GmbH + Co KG | ARBOCEL® |
| [34] | J. RETTENMAIER & SÖHNE GmbH + Co KG | ARBOCEL® RC FINE |
| [35] | Agromed Austria GmbH | OptiCell® |
| [36] | GLOBAL NUTRITECH® | LIGNOCHAR® |
| [37] | J. RETTENMAIER & SÖHNE GmbH + Co KG | ARBOCEL® FD00 |
| [38] | J. RETTENMAIER & SÖHNE GmbH + Co KG | ARBOCEL® R |
| [39] | J. RETTENMAIER & SÖHNE GmbH + Co KG | ARBOCEL® R |
| [51] | J. RETTENMAIER & SÖHNE GmbH + Co KG | ARBOCEL® RC FINE |
| [52] | J. RETTENMAIER & SÖHNE GmbH + Co KG | ARBOCEL® RC FINE |
| [53] | J. RETTENMAIER & SÖHNE GmbH + Co KG | ARBOCEL® RC |
| [69] | J. RETTENMAIER & SÖHNE GmbH + Co KG | ARBOCEL® RC |
| [70] | J. RETTENMAIER & SÖHNE GmbH + Co KG | ARBOCEL® R |
| [86] | Agromed Austria GmbH | OptiCell® |
| [96] | Agromed Austria GmbH | OptiCell® |
| [97] | unspecified | unspecified |
